# Predicting trajectories of vocational indecision from motivational profiles in early adolescence

**DOI:** 10.1186/s40359-024-01747-0

**Published:** 2024-05-03

**Authors:** Remy Mbanga, Catherine F. Ratelle, Stéphane Duchesne

**Affiliations:** 1 Department of Professional Guidance, Faculty of Education, Room A1-317, Sherbrooke University, Sherbrooke, QC J1K 2R1 Canada; 2 Department of Foundations and Practices in Education, Faculty of Education Sciences, Room 946, Laval University, Quebec City, Quebec G1V 0A6 Canada; 3 Department of Teaching and Learning Studies, Faculty of Education Sciences, Room 934, Laval University, Quebec City, Canada

**Keywords:** Vocational indecision trajectories, Chronic indecision, Motivational profile, Self-determination, Adolescence, Person-centered approach

## Abstract

**Background, objective and hypotheses:**

During emerging adulthood, vocational indecision (i.e., the inability to make coherent career choices) develops in a heterogeneous fashion, with three distinct patterns: low; decreasing (i.e., developmental or adaptative); high and stable or increasing (i.e., chronic or maladaptive). Among the determinants of vocational indecision that have been identified in past research, academic motivation is a crucial an excellent choice, since it is at school that students' vocational choices are validated or not. According to SDT, this motivation can vary both in quantity and quality, and students tend to experience more positive academic outcomes when their motivational profile is optimal (high quantity, high quality) as opposed to suboptimal (e.g., low quantity, low quality). Thus, the purpose of this longitudinal study was to verify if the patterns found with emerging adulthood students characterized vocational indecision in adolescent students, and if supported, to predict the belonging to the most problematic trajectory by using students’ academic motivational profiles. We expected several distinct trajectories of vocational indecision that would differ in shape and magnitude, and several motivational profiles that vary in quality as well as in quantity. We also expected students in high-quality or quantity motivational profiles to be less likely to follow a chronic indecision trajectory.

**Method and results:**

Using data from 384 students (56% female; Mage = 13.52 years; SD = .52 at Secondary 2) surveyed annually from Secondary 2 to 5, person-centered analyses enabled estimation of motivational profile in Secondary 2 and vocational indecision trajectories during the 4-year period. Results revealed four distinct patterns of vocational indecision during adolescence labelled Low and Stable, Moderate and Stable, Developmental and Chronic Intermittent. Four motivational profiles were also identified in Secondary 2, ranging from poor (Highly Amotivated) to moderate (Autonomous-Introjected) quality of self-determination level. Also, in reference to the most self-determined profile, students in the Mixed profile were at greatest risk of following Chronically-Intermittently Undecided trajectory. Finally, the most self-determined students were at greatest probability of following the Developmentally Undecided trajectory.

**Conclusion:**

Overall, the findings suggest that the student motivational functioning in early secondary school years could be used to identify students at risk of experiencing the negative indecision patterns across secondary school. Several theoretical and practical implications are suggested.

**Supplementary Information:**

The online version contains supplementary material available at 10.1186/s40359-024-01747-0.

Seeking, processing, and integrating vocational information is important for youth career decision-making and these developmental tasks can be carried with varying levels of difficulty. Indeed, while some youths go through these tasks rather easily, others will experience some ephemeral difficulties, or even substantial struggles [1–4], which leads to vocational indecision (i.e., the inability to make an educational and occupational choice [5]. Vocational indecision has its roots in the fear of making wrong choices or the difficulty of giving up alternatives [6–8]. It can lead students to experience psychological distress [6], dropping out of school [7] or opting for more precarious jobs that offer lower wages and disadvantageous conditions [6] (e.g., employment without a retirement plan) [8]. These issues justify the necessity to better understand what makes some students be more undecided than others. Using emerging adulthood samples (i.e., college students), previous studies showed that the expression of some vocational processes such as exploration or indecision, change over time [9, 10]. Precisely, these vocational processes were found to develop heterogeneously, as revealed by three main trajectories: stable, increasing, and decreasing [9, 10]. In fact, experiencing vocational indecision is normal, and it is not a detrimental phenomenon when it remains transitory (i.e., if it decreases after a successful processing of career-relevant information [2, 3]). This type of vocational indecision is labelled developmental [11, 12] and, while this developmental pattern can promote psychological distress, it is temporary and disappears once a career choice has been made [4]. In contrast, indecision becomes problematic when it is chronic or permanent (i.e., it increases over time or remains highly stable), because it generalizes to several spheres and leads individuals to experience permanently psychological distress (e.g., anxiety, nervousness) when having to make a decision [11, 13].

Most studies that distinguished chronic from developmental indecision used a cross-sectional design to categorize individuals into decided, developmentally undecided or chronically undecided [12, 14, 15]. This approach is suboptimal because it does not capture the temporal fluctuations in vocational indecision students experience during their schooling [16] and forces them into categories that might not represent their developmental process. Alternatively, using several measures over time allows grouping students according to their naturally occurring developmental pattern of vocational indecision. To do so, one must use a person-centered approach, which models interindividual differences to yield groupings such as profiles or developmental trajectory [17, 18]. Specifically, person-centered modelling of vocational indecision allows distinguishing between developmental and chronic vocational indecision. These developmental patterns were empirically supported in a sample of emerging adults, which was surveyed annually for 3 years [10]. Three trajectories were identified in a sample of 2300 students: high and increasing (chronically undecided; 25% of the sample), high and decreasing (developmentally undecided; 27%), and low and stable (decided; 48%). To our knowledge, no study has examined whether these heterogeneous patterns of vocational indecision can be observed during secondary school, which would allow identifying adolescents at greater risk of following chronic trajectory of vocational indecision. Doing so would help implement early and timely interventions to support their vocational development.

Also, it is important to note that, even if many vocational aspects are transitory during adolescence (due to students' high propensity to change or readjust as they develop), the chronicity of vocational indecision can still be observed at this stage of their development. In fact, some stable characteristics such as dispositional anxiety or neuroticism enhance youths’ risk to be chronically undecided [19]. Similarly, some motivational processes contribute to enhancing or reducing the risk of experiencing chronic or developmental indecision during adolescence. In fact, several studies suggest that students’ high-quality motivation (i.e., behaving in accordance with one’s authentic self) is linked to positive vocational development (i.e., they are more likely to follow developmental rather than chronic indecision trajectory). For example, while students following low or developmental indecision trajectories were found having a good quality of motivation, those following chronic indecision trajectory were found having a poor quality of motivation [10]. Also, students following a high trajectory of vocational exploration [9] (i.e., a protection factor against vocational indecision) were more likely to have their basic psychological needs met by their parents (which contributed to the quality of motivation). Finally, positive trajectories of vocational identity (negatively linked to vocational indecision) mostly included students reporting high-quality of academic motivation, while negative trajectories mostly included poor-quality of academic motivation [20]. Overall, these findings suggests that the quality of academic motivation appears to discriminate well students according to dimensions of their vocational development during the adolescence. As a result, student motivation was examined as a predictor of their vocational indecision trajectories. Our conceptualization of academic motivation was based on self-determination theory.

## Quality of student motivation

Self-determination theory (SDT) [21] is a humanistic theory of motivation that considers humans as proactive and naturally inclined to explore their environment in seeking well-being and optimal development (e.g., learning new things and developing one’s potential). A key feature of SDT is its distinction of types of motivation, which vary according to the underlying level of self-determination (i.e., their quality). First is intrinsic motivation, the most self-determined type of motivation, which is observed when one engages in a task because of the pleasure and satisfaction felt while doing it. This type of motivation contrasts with extrinsic motivation (EM), which is observed when individuals perform tasks for reasons other than the pleasure and satisfaction it brings them. There are four types of EM. The first is integrated regulation, which refers to engaging in a task because it integrates one’s value system and self. It is the most self-determined type of EM, but because it is difficult to measure in adolescent samples [21], it was not used in this study. The second type of EM is identified regulation, a self-determined type of EM that is observed when one engages in a task because it is personally important for them, and they deliberately choose to do it. The third type of EM is introjected regulation, where a task is carried out to reduce internal pressures (e.g., guilt) or for self-esteem concerns. Lastly, external regulation refers to engaging in an activity in response to outside contingencies such as to obtain a tangible reward or avoid punishment. These last two types of EM (external and introjected regulations) are controlled forms of motivation while intrinsic motivation and identified regulations are autonomous forms of motivation [21]. The last type of motivation within SDT is amotivation, manifested when individuals fail to see a valid reason for involving a task and lack purpose. This typology has been extensively validated and applied in several domains such as educational context [22], which is central to several vocational development processes [23, 24].

### Vocational development and academic motivation

First, it is important to distinguish youth vocational motivation (i.e., motivation to engage in the activities pertaining to career decision-making) from their academic motivation (i.e., motivation to engage in school). Indeed, vocational motivation is a more proximal predictor of vocational indecision than academic motivation, since vocational motivation and vocational indecision occur at the same level of generality [25, 26]. Yet, academic motivation includes several spheres of the school context (e.g., vocational, well-being, relationship), where many motivational mechanisms at the academic level (e.g., the quality of motivation) interact with some vocational processes [20, 23, 24]. This justifies our choice to focus on academic motivation to better understand students’ trajectory of vocational indecision.

In line with SDT, the relevance of focusing on academic motivation can be explained both theoretically and empirically. As students progress through school levels, they must make several academic choices related to school subjects (e.g., type of science or mathematics class), programs (e.g., sciences or literature) or extracurricular activities that have implications for their vocational development. The (in) stability of these choices has been found to affect their ability to make a vocational decision [27–29]. Theoretically, when students are volitionally going to school (i.e., are autonomously motivated), they are posited to make coherent or authentic academic choices (i.e., which concord with their aspirations and interests) [30], which as a results makes them more comfortable in making a career decision [3, 31]. Consequently, these students are more likely to be stable in their academic choices because they perceive the academic requirements as important steps to achieve rather than obstacles, and they have an easier time to adjusting as needed [21, 32]. This stability could allow students who are autonomously motivated for school to persevere in their orientation plan [33]. Therefore, they could be at lower risk of experiencing chronic vocational indecision and more likely to follow a developmental indecision trajectory.

In contrast, when students go to school for controlled motivations (e.g., to please their parents, so that their parents continue paying for their car, out of pride or guilt) or are amotivated, they are more likely to experience psychological distress such as persistent anxiety symptoms, discouragement and dissatisfaction with school, and academic failure [21, 34, 35]. To reduce the deleterious effects of these suboptimal motivations, controlled and amotivated students are more likely to make inauthentic or incoherent choices, because they are not guided by their own values, interests, or life aspirations [30, 36]. Eventually, these students are more likely to change their academic choices when difficulties or challenges arise, making them more liable to have unstable orientation plans and thus experience chronic indecision.

To our knowledge, no study has linked trajectories of vocational indecision with student motivational functioning in school. However, autonomous academic motivation, more than controlled, motivation and amotivation, has been associated with some vocational behaviours, processes, or mechanisms that optimize youth vocational development. Precisely, autonomous academic motivation has been positively associated with identity formation [20, 37], vocational exploration [24], and the nature of career choice (i.e., voluntary) [23]. In addition, emerging adults’ motivational functioning toward career decision-making predicts their vocational indecision trajectories, with decided students being the most autonomously engaged, compared to chronically undecided students [10].

Finally, academic amotivation was found to be stronger for students who explore their self and their environment the least [24]. Moreover, academic amotivation has been positively associated with the lack of concern for school, low aspirations, and absenteeism [38], school dissatisfaction and anxiety [34], and vocational indecision [39]. Thereby, amotivation, more than controlled academic motivation, could contribute to increasing vocational indecision. In conclusion, chronic vocational indecision could be exacerbated by controlled academic motivation and, more so, by academic amotivation. As all the three categories of academic motivation can simultaneously contribute to the expression of a process [40], some studies used a person-centered approach to identify how they combine within the self into motivational profiles, before linking them to educational outcomes [24].

### Academic motivation profiles in adolescence

As presented in Section S1 of Supplementary Materials, several profiles including autonomous motivations, controlled motivations, and amotivation were found in the educational context [22, 34, 41, 42]. Among these profiles, five recurrent groups were obtained with different secondary school samples, ranging either from poor to high self-determination or low to high quantity of all types of motivation:(1) a poor quality profile (Controlled-Amotivated, or Non-Self-Determined) is characterized by high amotivation, moderately high levels of controlled motivations, and low to moderate autonomous motivations; (2) a low quantity profile (Low Autonomous-Controlled) is characterized by low amotivation, autonomous and controlled motivations; (3) a moderate quantity and quality profile (Mixed) is characterized by moderately high to moderate levels of all types of motivation; (4) high quality profile (Autonomous or Self-Determined) characterizes students with low amotivation and controlled motivations, but high autonomous motivations; and (5) a high quantity profile (High Autonomous-Controlled), which entails moderate to low amotivation, but high levels of autonomous and controlled motivations.

In the educational context, high-quality (e.g., self-determined) and high-quantity (e.g., high autonomous and controlled motivations) profiles were associated with beneficial outcomes such as school satisfaction [34], academic performance [22, 41], and vocational exploration [24]. In contrast, low-quality (i.e., non-self-determined) and low-quantity (low autonomous and controlled motivations, and amotivation) profiles were linked to detrimental outcomes such as school anxiety [34] and low vocational exploration [24]. Thus, it could be that students with low-quality motivational profiles are more likely to follow a negative trajectory of vocational indecision (i.e., chronic). Conversely, students with high-quality motivational profiles would be more inclined to follow positive trajectories such as transient or adaptive indecision (developmental).”

## The present study

Three distinct trajectories of vocational indecision have been identified in emerging adulthood, with the most problematic being a chronic indecision trajectory. Although these trajectories were not examined during adolescence, their identification could allow for early detection of students at risk of following a chronic trajectory, when indecision has not crystallized. Consequently, three main objectives were pursued in this study.

### Objective 1

Estimate distinct trajectories of vocational indecision among secondary school students and assess whether they are homogeneous or heterogeneous. More than one trajectory of vocational indecision was expected, and these were posited to differ in shape (i.e., increasing, decreasing, stable) and magnitude (i.e., low, moderate, or high): low and stable (i.e., decided), decreasing (i.e., developmental undecided), and increasing or highly stable (i.e., chronic undecided).

### Objective 2

Estimate, at the beginning of secondary school, academic motivational profiles using autonomous motivations (i.e., intrinsic, and identified regulation), controlled motivations (i.e., introjected, and external regulations), and academic amotivation. More than one profile was expected, ranging from poor (i.e., non-self-determined) to good quality (i.e., self-determined), or from low (i.e., low levels of each of the five types of motivation) to high (i.e., high levels of autonomous and controlled motivation) quantity.

### Objective 3

Predict students’ membership into an indecision trajectory, especially the chronic pattern, based on their academic motivation profiles at the beginning of secondary school. We expected that, the more the quality or the quantity of motivational profile is, the less likely students are to follow a chronic trajectory of vocational indecision. Precisely, students with the most self-determined or with the highest quantity profile were expected to be at lower risk of following the most problematic trajectory of vocational indecision. In contrast, students with the non-self-determined or with the lowest quantity profile were expected to be at the greatest risk of following the most problematic trajectory of vocational indecision. In line with previous studies [9], these links were examined controlling for gender and Socio-Familial Adversity.

## Method

### Participants and procedure

Data came from a longitudinal study on student transition, adaptation, and persistence in school that surveyed adolescents annually for 6 years, from the end of primary school to the end of secondary school. In the Quebec education system, the pre-university curriculum consists of thirteen years of education: six years in primary school, five in secondary school, and two in college. Students’ vocational decisions begin at the end of Secondary 2, where students can opt for professional training (e.g., plumbing, hairdressing) or continue their studies in the general education pathway in Secondary 3. Data from Secondary 2 (Time 1; T1) to Secondary 5 (Time 4; T4) was used to estimate vocational indecision trajectories, while data from T1 was used to estimate motivational profiles.

After obtaining ethical consent from the approved committees, a stratified sample was generated by the Ministry of Education to be representative of Grade 6 students in public schools across the province of Quebec. Sampling was based on gender, geographic representation (rural or urban), and socioeconomic status. Parents were first contacted and, once they gave their consent, their child (i.e., students) were also asked to consent to participate. All families who agreed to take part in the study at T1 were contacted at subsequent measurement times, and a 5$ compensation was offered to each participant for each wave they participated in. Of the 787 families who participated in the study (i.e., once both consents obtained), the total sample includes 728 students (55% girls) who participated in at least one measurement time (each Spring), by filling a paper or web questionnaire (via a secure university server). This time lag is appropriate to estimate the changes that take place at various stages of vocational development during adolescence. Of these 728 students, 476 participated at T1, 437 at T2, 354 at T3, and 377 at T4. The exclusion criterion was the non-participation T1, as motivational profiles were estimated using data from the Secondary 2. As a result, the sample retained in the present study includes 384 students (56% female; Mage = 13.52 years; SD = 0.52) who participated at T1, and at least one other measurement time.

Most students (95%) reported French as their mother tongue and were born in Quebec (94%). About 41% of students’ mother reported an annual family income of 70,000 $ CAN or more, which compares to the average household income in Quebec at T1 (68,170 $ CAN). Also, 77% of mothers reported having at least a secondary school diploma.

## Measures

To measure *vocational indecision*, the Career Decision Profile (CDP) [43] was used. It included 16 items, divided into six subscales: decidedness (2 items; e.g., “I have decided on the occupation I want to enter (for example, electrical engineer, nurse, or cook)”, comfort (2 items; e.g., “I’m not worried about my career choice”), self-clarity (3 items; e.g., “I need to have a clearer idea of my abilities, my major strengths, and weaknesses”), knowledge about occupations and training (3 items; e.g., “do not feel I know enough about the occupations that I am considering”), decisiveness (3 items; e.g., “I frequently have difficulty making decisions”), and career choice importance (3 items; e.g., “My future work or career is not that important to me right now”). For each item, participants indicated their level of agreement using an 8-point Likert scale (1 = not at all; 8 = totally). After performing measurement models, only 15 of the 16 items of the CDP were used as one item of the Comfort dimension behaved poorly. The 15 items were averaged to represent vocational indecision, which presented satisfactory psychometric qualities of the CDP (ω_T1-T4_ = 0.71 to 0.82), in line with past studies [44, 45] that supported its reliability (α = 0.89 and 0.88).

The Academic Motivation Scale (AMS) [46] was used to assess *academic motivations* at T1. It included 20 items assessing 5 types of motivation (4 items each), by asking students to indicate why they go to school. Each item represented a type of motivation: intrinsic motivation (e.g., “Because I experience pleasure and satisfaction while learning new things”), identified regulation (“I believe that a few additional years of education will improve my competence as a worker”), introjected regulation (e.g., “I want to show myself that I can succeed in my studies”), external regulation (e.g., “In order to obtain a more prestigious job later on”), and amotivation (e.g., “I can’t see why I go to school and frankly, I couldn’t care less”). For each item, participants indicated their level of agreement using a 5-point Likert scale (1 = not at all; 5 = completely), and the mean of the 5 items constituted the student’s motivational score. The psychometric qualities of the AMS were satisfying: ω = 0.92 for intrinsic motivation, ω = 0.76 for identified regulation, ω = 0.85 for introjected regulation, ω = 0.80 for external regulation, and ω = 0.82 for amotivation. They were similar to that of past studies (ω = 0.83 for intrinsic motivation, ω = 0.71 for identified regulation, ω = 0.71 for introjected regulation, ω = 0.73 for external regulation, and ω = 0.83 for amotivation) [46].

Students also reported their gender and age, while their mother reported on her education level, family income, and marital status. These three indicators were used to estimate the Socio-Familial Adversity Index (SAI) [47], used to control the socioeconomic status in a parsimonious way, by estimating if students were “at risk” or “not at risk” to experience family adversity. The SAI is a parsimonious indicator of socioeconomic status, combining parental income, education level, and family status, which was found to contribute to some vocational processes (e.g., exploration; Gagnon et al., 2019). The SAI has been used in studies that examined adolescents’ academic functioning [48] and vocational processes (e.g., exploration [9]). In this study, students were considered as being “at risk” (1) when their mother did not earn a secondary school diploma (23% of the sample), (2) they came from a family whose annual income was below 29,000 $ CAD (17%), and (3) whose family structure was not intact (i.e., parents separated, divorced, or widowed; 30%). Thereby, the SAI showed that 51% of the sample could be considered not at risk, 42% at low to moderate adversity risk, and 7% could be considered as highly at risk.

## Statistical analyses

### Preliminary analyses

After ensuring that data satisfied basic statistical postulates (e.g., normality, homogeneity of variances), the measurement qualities of vocational indecision and each type of motivation were examined (see Section S2 of Supplementary Materials). Next, the longitudinal invariance was tested to ensure that the measurement of vocational indecision was stable across the four waves. Based on measurement models, factor scores (i.e., standardized values whose mean is 0 and standard deviation is 1) rather than observed means were used, because they permit the partial control of measurement errors and preserving the underlying structure of the measurement model when testing complex models [49]. Vocational indecision's factor scores were saved from the configural invariance measurement model [50], while those of each type of motivation were saved from the confirmatory factor analysis measurement model.

### Trajectories of vocational indecision

The General Growth Mixture Analysis (GGMA) is a person-centered approach that allows the estimation of latent trajectory classes marked by different average shapes while including within-class variability [51]. The GGMA is conducted in two main steps [52]. In the first step, an unconstrained model is estimated, where intercept and slope are free to vary: it is the GGMA Mplus Default (GGMA-MD). If GGMA-MD solutions are improper (e.g., presence of negative variance), then the constrained model, the GGMA Latent Variable (GGMA-LV), is tested. In GGMA-LV the intercept and slope are fixed to 0 in the first class and freely estimated in subsequent classes [51, 52].

### Motivational profiles

Latent Profile Analysis (LPA) [52] was conducted to estimate academic motivation profiles using intrinsic motivation, identified, introjected and external regulations, and amotivation. LPA is a mixture analysis that enables the identification of underlying groups within a population and estimates the probability that individuals belong to these groups [53]. Residual variances of each type of motivation were constrained to equality across the classes, to ensure stable links across profiles [54]. It should be noted that motivational profiles were estimated at Secondary 2 and not at Secondary 1, to lessen the instability of motivational functioning related to the transition from primary to secondary school.

### Model estimation and retention

Different models with different groups were estimated and compared under GGMA and LPA, aiming to determine which one fits the data best. Models were estimated using Mplus [55] (version 8.3), and missing data were handled with the full information maximum likelihood (FIML) estimator, which is preferable to listwise deletion or other imputation methods (e.g., EM algorithm, mean substitution) [54]. Model retention in LPA and GGMA was based on the lowest Bayesian Information Criterion (BIC), Sample-Adjusted BIC (SABIC), and Akaike’s Information Criterion (AIC) [56]. In addition, the Vuong-Lo-Mendell-Rubin-likelihood ratio test (VLMR), the Lo Mendell-Rubin adjusted LRT test, (LMRA) and the Bootstrapped likelihood ratio test (BLRT), were used to determine best-fitting models [57]. Hence, even if the values for BIC, SABIC, and AIC of the (*N)-*class model were lower than those of the (*N-1)*-class model, the former was considered superior only if it presented a statistically significant *p* value on VLMR, LMRA, and LRT, suggesting that the addition of one group improved the parsimony of the (*N)-*class model [57]. The entropy (i.e., the degree to which individuals are well classified, with minimal uncertainty) was also estimated, and a model classification was considered acceptable when entropy was 0.80 or above [54].

### Predicting trajectory membership from motivational profiles membership

The Latent Transition Analysis (LTA) [52] was used to determine if students’ motivational profile membership predicted vocational indecision trajectory they belonged to. The LTA permits to determine the predictive links between two or more mixture sub-models (e.g., LPA and GGMA) within a single general model by using multinomial regression [58]. Specifically, it uses a reference class in each sub-model to estimate the unstandardized coefficients (B) corresponding to the logit of odds ratios (OR) (see Section S4 of Supplementary Materials). So, the LTA permitted to determine whether, belonging to a particular motivational profile rather than the most self-determined (i.e., the reference group in LPA) at T1, increased or decreased the risk of following a specific vocational indecision trajectory, rather than the most problematic (i.e., the reference group in GGMA).

### Covariates analyzes

After estimating the most optimal LPA and GGMA (without any control variable), we used the auxiliary approach to verify whether motivational profiles and vocational indecision trajectory groups were distinguished by gender and SAI or predicted by them. If they were found to predict motivational profiles or trajectory groups, the control-LPA and control-GGMA (i.e., including gender and SAI) were used as sub-models in the LTA (i.e., the predictive model of vocational indecision trajectories from motivational profiles).

## Results

### Preliminary analyses

Data screening revealed no violation of basic statistical assumptions. The optimal measurement model for vocational indecision was a second-order exploratory structural equation modeling within confirmatory factorial analysis, and it presented a metric invariance over time (see Section S3 of Supplementary Materials). Bivariate analyses showed that assumptions of the person-centered approach (i.e., independence of observations, absence of multicollinearity between motivational variables, and vocational indecision) were met, thus enabling to perform logistic analyses [59]. The correlations were at best weak, where vocational indecision was negatively associated with intrinsic motivation and identified regulation, and positively associated with external regulation and amotivation (see Table 1). Nevertheless, these links appeared stronger in person-centered analyses when trajectories of vocational indecision were associated with motivational profiles.

**Table 1:** Correlations among vocational indecision, types of motivation, and sociodemographic variables (*N* = 384)

	1	2	3	4	5	6	7	8	9	10	11
1. Vocational Indecision at T1^a^	‒										
2. Vocational Indecision at T2^a^	.68^*^	‒									
3. Vocational Indecision at T3^a^	.54^*^	.67^*^	‒								
4. Vocational Indecision at T4^a^	.59^*^	.62^*^	.72^*^	‒							
5. Intrinsic Motivation at T1^b^	-.06	-.14^*^	-.10	-.09	‒						
6. Identified Regulation at T1^b^	-.02	-.10	-.06	-.09	.42^*^	‒					
7. Introjected Regulation at T1^b^	.07	-.06	-.05	-.09	.54^*^	.35^*^	‒				
8. External Regulation at T1^b^	.14^*^	.03	.05	.09	-.12	-.41^*^	.17^*^	‒			
9. Amotivation at T1^b^	.08	.11	.15^*^	.19^*^	-.06	-.46^*^	-.19^*^	.03	‒		
10. Gender	.03	.02	.01	.01	.03	.08	.06	-.11	-.20	‒	
11. Sociofamilial Adversity Index^c^	.01	-.07	-.06	.01	-.08	-.05	.04	.03	.12	-.02	‒
*M*	4.15	4.12	4.17	3.44	3.33	4.37	2.94	3.89	1.45	1.56	.25
*SD*	1.62	1.66	1.71	1.68	1.06	.66	1.13	.90	.76	.49	.32

^⁎^*p* < .05

^a^Used an 8-point scale

^b^Used a 5-point scale

^c^Higher scores indicate stronger adversity

### Estimating trajectories of vocational indecision

**Fig. 1:**
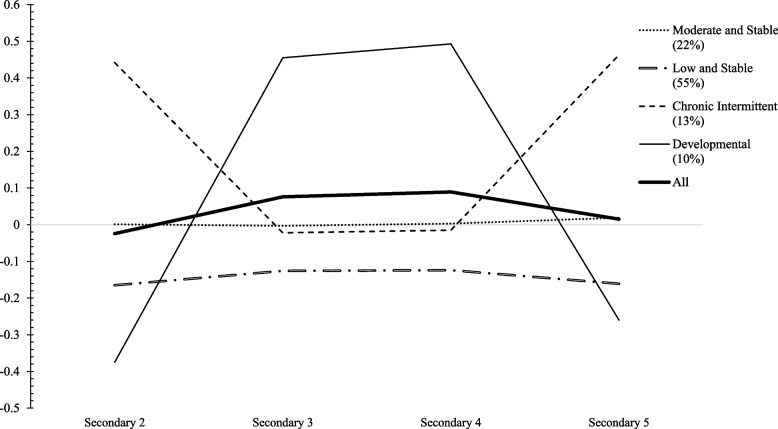
Trajectories of vocational indecision (*N* = 384). Note. Factor scores were used, with a mean of 0 and a standard deviation of 1

Fit indices suggested that the most optimal GGMA model was a 4-group GGMA-LV model (see Model 1, the unconditional GGMA-LV on Table 2). Because auxiliary analyses revealed that the probability to belonging in one group rather than another is predicted by SAI and student gender (see Section S3 of Supplementary Materials), both variables were included as control variable in Model 1 and allowed to estimate the control-GGMA-LV (see Model 2 on Table 2). The most optimal Model 2 was a 4-group model. Precisely, four distinct and heterogeneous developmental patterns of vocational indecision were found (see Fig. 1). The first was labelled the *Moderately High and Stable* trajectory (22% of the sample) in which vocational indecision remained high and stable over time. The second was the *Low and Stable* trajectory (55%), magnitude was below the sample average and remained stable across the 4 years. The third was the *Developmental* trajectory (10%), characterized by low levels of indecision at T1, which exponentially increased until T2 and remained very high until it exponentially decreased from T3 to T4. Finally, the fourth was the *Chronic Intermittent* trajectory (13%), characterized by the opposite pattern: very high levels of indecision at T1, which exponentially decreased between T1 and T2 and remained moderately stable until T3, followed by an exponential increase from T3 to T4. The chronic trajectory was considered to be the most problematic because it presented features of increasing of vocational indecision at the end of secondary school, reflecting persistent difficulties in the decision-making process when students must transition out of secondary school and make important vocational decision. Quadratic parameters were statistically significant for Developmental and Chronic Intermittent trajectories, with moderate effect sizes, which were respectively positive and negative (see Section S3 of Supplementary Materials).

**Table 2:** Fit indices for all models (*N* = 384)

		Estimates					*p* value		
Models	N by group	-2LL	**AIC**	BIC	SABIC	E	VLRM	LRMA	BLRT
GGMA-LV									
M1: Unconditional									
3 groups	65;95;224	-1042.04	2126.07	2209.03	2142.40	.75	.02	.02	.00
4 groups	37;51;85;211	-982.10	2021.97	2132.59	2043.75	.79	.01	.01	.00
5 groups	24;28;48;84;200	-964.95	1999.90	2138.17	2027.12	.75	.09	.09	.00
M2: Control									
3 groups	64;98;222	-1037.36	2124.72	2223.48	2144.16	.75	.20	.20	.00
4 groups	31;61;88;204;	-973.16	2014.32	2040.76	4263.81	.80	.02	.02	.00
5 groups	25;28;51;87;193	-954.51	1995.01	2164.89	2028.46	.75	.08	.08	.00
LPA									
M3: Unconditional									
2 groups	37;347	-1635.42	3302.84	3366.05	3315.29	.96	.08	.08	.00
3 groups	20;51;313;	-1555.14	3154.28	3241.20	3171.39	.96	.24	.26	.00
4 groups	18;45;94;227	-1499.07	3054.14	3164.76	3075.92	.82	.05	.05	.00
5 groups	15;36;43;80;210	-1455.60	2979.20	3113.52	3005.64	.85	.51	.51	.00
M4: Control									
2 groups	38;346	-1633.08	3302.17	3373.28	3316.17	.95	.25	.25	.00
3 groups	23;51;310	-1544.86	3141.72	3244.43	3161.94	.96	.65	.65	.00
4 groups	18;45;92;229	-1489.79	3047.58	3181.91	3074.03	.82	.00	.00	.00
5 groups	18;33;41;73;219	-1445.95	2975.91	3141.83	3008.57	.84	.37	.37	.00
Control LTA									
M5	‒	-2606.96	5319.93	5512.35	5361.15	.86	‒	‒	‒

Sex and SAI were controlled in all the control models. M5 = models 5a, 5b and 5c had the same fit indices

*p* *p*-value, *GGMA-LV* general growth mixture analysis-latent variable, *LPA* latent profile Analysis, *LTA* latent transition analysis

### Identifying academic motivational profiles

**Fig. 2:**
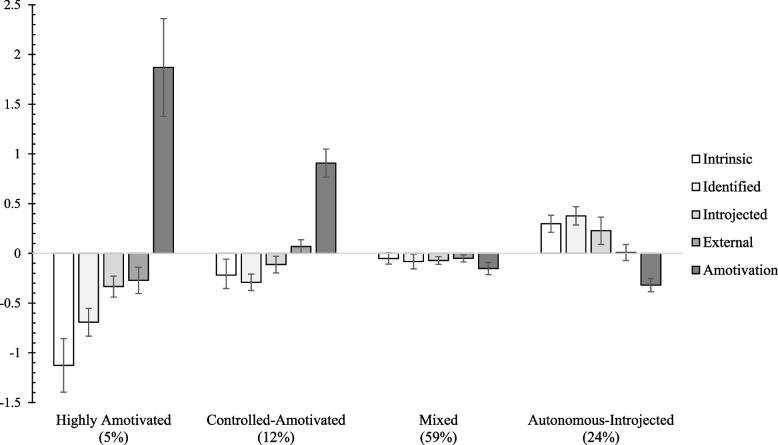
Motivational profiles in secondary 2 (*N* = 384). Note. Factor scores were used, with a mean of 0 and a standard deviation of 1

Results suggested that the most optimal LPA model was a 4-group solution (see Model 3, the unconditional-LPA on Table 2). As with vocational indecision trajectories, auxiliary analyses revealed that the probability to belonging in one group rather than another is predicted by SAI and student gender. So, using Model 3, student’s gender and SAI were included as predictors of profile membership (see Model 4, control-LPA on Table 2). The most optimal Model 4 was a 4-group model, and the four profiles showed different levels and patterns on each type of motivation (see Fig. 2). The *Highly Amotivated* profile (5% of the sample) included students reporting very low levels of intrinsic motivation and identified regulation, low levels of introjected and external regulations, but very high levels of amotivation. The *Controlled-Amotivated* profile (12%) was characterized by low levels of intrinsic motivation and identified regulation, moderate introjected and external regulations, but high amotivation. The *Mixed* profile (59%) presented moderate levels of all the five motivations. The *Autonomous-Introjected* profile (24%) included students reporting highly moderate levels of intrinsic motivation, identified and introjected regulations, moderate levels of external regulation, and a low level of amotivation. This last profile was considered as the most self-determined and used as the reference group in LTA.

### Associations between trajectories of vocational indecision and motivational profiles

The LTA model that was used to predict trajectories of vocational indecision from motivational profile presented satisfactory fit indices (see Model 5 in Table 3). Regarding the distribution of students from each profile into each trajectory, four observations can be made. First, most students in each motivational profile followed the Low indecision trajectory: 59% from the Controlled-Amotivated profile, 58% from the Mixed, 50% from the Highly-Amotivated, and 42% from the Autonomous-Introjected profile. Second, the Moderate and Stable trajectory was followed by 33% of students in the Autonomous-Introjected profile, 28% in the Highly-Amotivated profile, 27% in the Controlled-Amotivated profile, and 18% in the Mixed profile. Third, the Chronic Intermittent trajectory was followed by 22% of students in the Highly Amotivated profile, 18% in the Mixed profile, 8% in the Autonomous-Introjected profile, and 5% in the Controlled-Amotivated profile. Finally, the Developmental trajectory was followed by 17% of students in the Autonomous-Introjected profile, 9% in the Controlled-Amotivated profile, 6% in the Mixed profile, but none of the students in the Highly Amotivated profile. Details of this distribution are provided in Section S4 of Supplementary Materials.

**Table 3:** Prediction of vocational indecision trajectories membership by motivational profiles membership

	Vocational indecision trajectories		Estimates					
Motivational Profiles	Reference Trajectory	Targets Trajectories	B	SE	*p*	[95% CI]	OR	% of Change
Model 5a: Highly Amotivated (*vs* Autonomous-Introjected)	Chronic Intermittent	Low and Stable	-.82	1.05	.43	[-2.54, .90]	.44	↓55%
		Moderate and Stable	-1.02	1.08	.34	[-2.79, .75]	.36	↓63%
		Developmental	‒	‒	‒	‒	‒	‒
Model 5b: Controlled- Amotivated (*vs* Autonomous-Introjected)	Chronic Intermittent	Low and Stable	.59	1.06	.58	[-1.16, 2.34]	1.80	↑80%
		Moderate and Stable	.03	1.19	.99	[-1.92, 1.99]	1.03	↑3%
		Developmental	.10	1.99	.96	[-3.17, 3.36]	1.11	↑11%
Model 5c: Mixed (*vs* Autonomous-Introjected)	Chronic Intermittent	Low and Stable	-.35	.80	.67	[-1.66, .97]	.70	↓30%
		Moderate and Stable	-1.35	.81	.10	[-2.68, -.02]	.26	↓74%
		Developmental	-1.63	1.04	.12	[-3.34, .08]	.20	↓80%

↓and ↑ = increase and decrease in % change

*p p* value, *B* unstandardized regression coefficient, *SE* standard error, *CI* confidence interval, *OR* odd ratio

For multinominal regressions, the Autonomous-Introjected profile (i.e., the most self-determined) and the Chronic Intermittent trajectory (i.e., the most problematic) were used as reference groups in LTA. So, OR indicated whether belonging to one of the three motivational profiles, rather than the Autonomous-Introjected profile, increased (values above 1) or decreased (values below 1) the probability of following the Chronic Intermittent trajectory, rather than the Developmental, Low and Sable or Moderate and Stable trajectories. Thereby, using Model 5, three different predictive sub-models were tested in reference to the Autonomous-Introjected profile: Model 5a (Highly-Amotivated *vs* Autonomous-Introjected), Model 5b (Controlled-Amotivated *vs* Autonomous-Introjected) and Model 5c (Mixed *vs* Autonomous-Introjected). In line with results of auxiliary analyses, SAI and gender were controlled in each of the three LTA models (i.e., 5a, 5b and 5c).

Results of Model 5a showed that being in Highly Amotivated profile increased students’ probability of following the Chronic Intermittent trajectory rather than any other trajectory, while students with this profile were at the lowest probability of following the Low and Stable trajectory as well as they could not follow the Developmental trajectory. Results for Model 5b showed that being in the Controlled-Amotivated profile decreased students’ probability of following a Chronic Intermittent trajectory rather than any other one, while students with this profile were at the greatest probability of following the Low and Stable trajectory. Results for Model 5c showed that being in the Mixed profile increased students’ probability of following a Chronic Intermittent trajectory rather than any other one, while students with this profile were at the lowest probability of following the Developmental trajectory. In addition, students with a Mixed profile, more than those with a Highly Amotivated profile, were at greatest risk of following a Chronic Intermittent trajectory. Consequently, students with an Autonomous-Introjected profile were not at the lowest risk of following a Chronic Intermittent trajectory, neither at the greatest probability of following a Low and Stable trajectory. But they were at the greatest probability of following the Developmental trajectory.

## Discussion

### On trajectories of vocational indecision during adolescence

#### Overview of results

In line with our expectations, the development of vocational indecision status was found to be heterogeneous and characterized by four distinct trajectories: Low and Stable, Moderate and Stable, Developmental, and Chronic Intermittent. These findings demonstrate how the development of vocational indecision occurs in a heterogeneous fashion during adolescence. In fact, although several studies have conceptualized vocational indecision as a dynamic process, most of them did not use a longitudinal design, such that the classification of people into decided, developmentally undecided, and chronically undecided categories relied solely on indecision scores from a single data wave [11, 14, 60]. This constitutes an important limitation when assessing the expression of a time-varying process such as vocational indecision [61]. To our knowledge, only one longitudinal study examined vocational indecision trajectories, but it focused on emerging adults [10]. Here, using longitudinal data from secondary 2 to 5, we replicated the three theoretical indecision patterns previously obtained in emerging adults (i.e., low, chronic, and developmental), which suggests that the heterogeneous development of vocational indecision begins during adolescence.

#### Main findings and implications

Our results present three main points. First, contrary to conventional wisdom that vocational indecision is only transitory in adolescence, this study shows that adolescents can develop chronic indecision. Hence, although adolescence is a developmental stage during which indecision is normal, some students appear to crystallize their indecision until it becomes chronic. It is therefore important to identify these students early, to reduce their risk of becoming chronically undecided. Second, four developmental trajectories were obtained with adolescents, compared to three with emerging adults [10]. The fourth trajectory identified in adolescents is characterized by moderate and stable levels of vocational indecision throughout the 4-year period and included a larger proportion of students than chronic and developmental trajectories together. Given the large proportion of students following this trajectory and knowing that most secondary school students go on to university in Quebec, this pattern could indicate that students’ involvement in their decision-making is normative at this stage of their schooling. They could settle for a minimal investment in time and energy and wait until the beginning of college to begin more actively making career-relevant decisions. This delay could result from the endorsement of nonproductive coping strategies such as escape, that leads students to make random or unreflected choices, because they intend to change them later in their schooling [62]. Third, whereas chronic and developmental trajectories followed a linear trend in emerging adults, these trajectories revealed quadratic effects during Secondary 3 and 4, implying that these school years correspond to critical periods in adolescents’ vocational decision-making. Fittingly, this corresponds to students’ obligation to make academic choices during this period (e.g., mathematics option in Secondary 3, the type of science course in Secondary 4). Vocational indecision could therefore be amplified for some students at this period, while others could be more comfortable in such vocational decision-making.

### On motivational profiles

#### Overview of results

As expected, more than one motivational profile was obtained, varying from very poor to moderate quality: Highly Amotivated, Controlled-Amotivated, Mixed, and Autonomous-Introjected. These findings contribute to the motivational literature, by replicating some of the motivational profiles obtained in past studies with secondary school and college students: the Controlled-Amotivated profile (i.e., poor quality) [63], the Mixed profile (moderate quantity and quality) [34], and the Autonomous-Introjected profile (i.e., moderate quality) [22]. In addition, similarly to past studies with secondary school students, we did not identify a purely autonomous profile (i.e., students expressing high levels of autonomous motivations and low levels on all other types of motivation). In fact, this profile was rarely identified in youth entering in secondary school (vs. college level; see Section S1 of Supplementary Materials). One reason might be the fact that, when beginning secondary school, students are not yet used to the many external and internal pressures in school (e.g., academic rules, identity conflicts), which could fuel their controlled motivations.

#### Main finding and implication

In addition to these results, which corroborate those of previous studies, we found that, motivational profiles characterized by high amotivation also included students who reported moderate to high levels external regulation in secondary school (see Table 1). But in line with past results obtained with adult samples [64], adolescents in the Highly Amotivated profiles could express strong levels of amotivation, concomitantly with very weak levels on other forms of motivation.

### On the prediction of trajectories of vocational indecision from motivational profiles

Predicting students’ membership into the most problematic trajectory vocational indecision (i.e., Chronic Intermittent) from academic motivational profiles membership, our third hypothesis was partially supported. Specifically, students’ motivational profiles at the beginning of secondary school allowed identifying those at higher risk of following this maladaptive pattern. But as the expected predictive links were not all supported, all these findings are unique.

#### Overview of results

First, students with the least self-determined profile (i.e., Highly Amotivated) were not at the greatest risk of following the chronic trajectory, contrary to our expectations, and students with the most self-determined profile (i.e., Autonomous-Introjected) were not at the lowest risk of following it. In fact, because of their moderately high level of autonomous motivation, students with an Autonomous-Introjected profile must engage voluntarily and authentically in school, leading them to adopt several positive vocational behaviours (e.g., vocational exploration or identity consolidation) which contribute to reducing vocational indecision. This could explain why these students, more than those from any other profile, followed the Developmental trajectory, suggesting their greatest capacity to resolve vocational conflicts [3]. However, the moderate level of introjected regulation in this profile indicates that internal pressures (e.g., feel obliged to meet academic requirements) [24] also operate. These pressures could lead students to make inauthentic academic choices, as suggested by their greater probability of following Moderate trajectory. So, some students with an Autonomous-Introjected profile are minimally involved in their decision-making process and postpone formulating a coherent and authentic choice.

Second, the same level of all the five types of motivation in students with a Mixed profile suggests that they are experiencing substantial motivational conflicts. That is, they go to school for concomitant reasons: pleasure to learn, importance of studies, reduction of internal and external pressures, avoidance of punishment or receiving of reward, or without purpose. These motivational conflicts could justify the high level of indecision at the beginning of secondary school, in students with this profile. However, it would be possible that controlled motivations guide students' choices at critical points in their schooling (Secondary 3 and 4), when they are required to make a choice in mathematics and science. Therefore, students make choices that does not necessarily correspond to their aspirations, hence a reduction in their indecision during this period. But, at the end of secondary school, these inauthentic choices are questioned again, hence a rise in their indecision. Also, because students in the Mixed profile were at greatest risk of following the chronic indecision trajectory, it seems that motivational conflicts constitute a noteworthy risk factor for chronic indecision. It is worth noting that individuals with a conflictual career indecision profile (characterized by high internal and external conflicts) were found to perceive most career decisional as destressing, more than those with an amotivated profile (i.e., lack of motivation) [65]. This could also justify why students with a Mixed profile were more prone to chronic indecision, compared to those with a Highly Amotivated profile.

Third, students with a Highly Amotivated profile were at greater risk of following a chronic indecision trajectory, rather than any other trajectory. Academic amotivation is associated with low engagement in school activities and tasks, weak academic achievement, low self-efficacy, disregard for school, and underidentified vocational interests [38]. These negative perceptions and behaviors could alter students’ capacity to seek or integrate vocational information, leading to their greater risk to be chronically undecided. In addition, the fact that none of these students followed the Developmental trajectory implies that they were unable to resolve their vocational difficulties in contrast to students from any other profile, especially in the Autonomous-Introjected profile. This could be explained by their very weak levels of autonomous and controlled motivation, reflecting their lack of intentionality (either autonomous or controlled).

Fourth, students with a Controlled-Amotivated profile were less likely to follow the Developmental trajectory, compared to those with an Autonomous-Introjected profile. This suggests that they struggle more when faces with vocational challenges in one or several vocational mechanisms (e.g., collection or integration of vocational information) or processes (e.g., exploration, identity formation), or experience difficulties at a personality level (e.g., trait anxiety). In fact, as their motivational functioning is qualitatively poor, students with a Controlled-Amotivated profile are posited to make inauthentic academic choices, which are not line up with their values, interests, life aspirations, future-oriented goals, and decisions [30]. So, students’ academic choices are related to the wanting to protect themselves from internal (e.g., anxiety) or external (e.g., being forced to make a course selection) pressures, not being separated from friends, receiving reward, or wanting to please parents [35]. In other words, the decreasing of vocational indecision in this group could be transitory or ephemeral. However, as students with a Controlled-Amotivated profile were at lowest risk of following chronic intermittent trajectory, and at greatest probability of following the low trajectory, it seems that the reduction of vocational indecision in secondary school is mostly guided by controlled motivations. This corroborate our findings with the Mixed and Autonomous-Introjected profiles, where controlled motivations were posited to prone the making career-decision.

#### Implications

These results suggest that when students experience academic motivational conflicts, high amotivation or high introjected regulation, they are more likely to be chronically undecided. This highlights the importance of identifying such students early in secondary school. Therefore, for students expressing motivational conflicts at the beginning of secondary school (e.g., those with a Mixed profile), guidance counselors could help them prioritize what they really or authentically like in school. This would help them better identify and consolidate their vocational aspirations or interests, and consequently decrease their likelihood to be experiencing career decision-making difficulties. For students whose profile combines high levels of amotivation and low levels on other types of motivation, guidance counselors could help them identify the importance of school for developing their skills and learning content, which will be relevant in a technical job (e.g., plumber, electrician). This will increase students’ perception of the school path as being relevant to their future career rather than a burden. Also, customed adjustments could be offered to amotivated students who experience academic difficulties and want to leave school for a professional training. For instance, as developing a schedule where manual and intellectual activities alternate (e.g., work-study, on-the-job training).

Education stakeholders need to pay special attention to students who go to school to relieve internal pressures (even if they concomitantly present autonomous forms of motivation), as our findings show that the benefits associated with autonomous motivation are undermined by introjection, by increasing their probability to be chronically undecided. Therefore, it is important to intervene for reducing these students’ internal pressures, by supporting their need for autonomy in school. In this perspective, professionals could create an environment where students direct the activities they do in class, so they could choose tasks that align with their priorities and capabilities [66]. Professionals could also align their task to students’ values, by explaining to them the reason to perform a behaviour or doing an activity [66]. These actions would increase students’ autonomous motivation in school; therefore, they would really cater for their decision-making process in secondary school, rather than escaping the whole process of or postponing a choice (i.e., until college).

#### Theoretical implications

Findings from this study provide further empirical support for the relevance of adopting a person-centered approach when studying vocational indecision. The bivariate correlations showed very weak associations between vocational indecision and each type of academic motivation, which indicates that these processes are unrelated. Adopting a variable-centered approach (i.e., considering adolescents as a homogeneous group) therefore conveys that their academic motivation has no bearing on students’ vocational decision-making. In contrast, observing these links through a person-centered approach – both for academic motivation (using motivational profiles) and vocational indecision (using trajectories) – indicate otherwise. Specifically, within-person combination of academic motivations in early adolescence explains which developmental pattern of indecision they will follow until the end of adolescence.

### On gender and socio-familial adversity

Our results indicate that students with a high Socio-Familial Adversity Index (SAI) and boys, are most at risk of following a chronic trajectory of vocational indecision and present poor motivational quality profile (See Sect. 3 in the Online Supplementary Materials). So, practitioners should pay attention to students’ gender and socioeconomic adversity, since youths who experience greater adversity in their family (as per the SAI score) were most at risk of following a chronic trajectory of indecision and being unmotivated (and therefore most at risk of leaving school early); this risk is further exacerbated among boys.

### Strengths, limits and future research

This study has several strengths, such as the use of a stratified sample from the Ministry of Education, a longitudinal design, sophisticated analyses, and the examination of the predictive validity of academic motivations using a person-centered approach. However, it has several limitations that can nuance the interpretation of our findings. First, our sample was strongly homogeneous: mostly Caucasian, born in Quebec, and speaking French, which could affect the generalization of our results to other contexts and students. Future studies linking adolescents’ motivation and career decision-making should have greater heterogeneity of participants in terms of race, origin and first language. Second, only students’ perspective was considered, even though other key stakeholders within schools contribute to youth vocational development such as teachers, guidance counselors, and so on. This increase bias due to shared method variance, an issue that can be remedied in future research by considering the stakeholders perspective not only in the measure of vocational indecision, but also in that of students’ academic motivation. Third, our design was descriptive (i.e., not experimental control was exerted on our variables) and does not allow the drawing of causal inferences. So, we cannot confirm that vocational indecision trajectory membership is a consequence of academic motivational profile membership.

Importantly, another avenue is to ascertain the chronicity of vocational indecision, by linking each trajectory with some vocational processes which have been found to discriminate the chronic to not chronic pattern. For example, chronic pattern of vocational indecision was strongly associated with low career aspirations, interests, educational intentions and well-being, and high anxiety (anxiety-trait). Using an auxiliary approach, it would be possible to discriminate trajectories of vocational indecision according to these processes, then confirm the chronicity of vocational indecision. Also, the temporal stability of motivational profiles across secondary school could be considered in future research. Precisely, students can switch from one motivational profile to another, and that new profiles can emerge from one grade to another during secondary school. So, future studies could used other time points (e.g., Secondary 3 or 4) to predict vocational indecision trajectories membership, then comparisons of predictive effects could be made according to the similarity of motivational profiles between two different measure times. In addition, it will be useful to determine if the transition from one motivational profile to another (e.g., from Secondary 2 to 3) modify the students’ probability of belonging to a vocational indecision trajectory followed in the precedent grade.

## Conclusion

The focus of this study was to determine if chronic pattern of vocational indecision take place since the adolescence, and if so, predict the membership into this problematic trajectory, using students’ academic motivation profiles at the beginning of secondary school. This study demonstrated the presence of four developmental patterns of vocational indecision into adolescents, among which, the chronic trajectory. It would seem that, when students are going to school for concomitant reasons such as the pleasure to learn or develop themselves, the importance of studies, the wanting to reduce internal and external pressures, to avoid punishment, to receive a reward, or without any purpose, they are at greatest risk to develop the chronicity of indecision. Therefore, in the aim to prevent the following of chronic trajectory of vocational indecision in adolescence, students must early identify the purpose of why they are going to school, even if it is related to suboptimal forms of motivation, which could be addressed by some adapted self-determined interventions [66]. This leads to better identify, focus, and crystallize vocational interests and aspirations, which would allow students to easily make vocational choices, and diminish their vocational indecision [30]. This study presents several methodological strengths, as the using a person-centered approach (motivational profile and trajectories of vocational indecision) and a longitudinal design, and a strong theoretical grounding using self-determination theory. However, future studies are encouraged to replicate these findings using homogenous samples, and or in other contexts (i.e., out of Quebec). Also, future studies must better investigate the chronicity of vocational indecision, using some vocational mechanisms such as anxiety or neuroticism, that have been empirically found to characterize chronic indecision [19].

### Supplementary Information

**Supplementary Material 1.**

## Data Availability

This manuscript does not report data generation or analysis. The data that support the findings of this study are available from the corresponding author upon request.
