# Supplementary material for: Predicting trajectories of vocational indecision from motivational profiles in early adolescence
Source: BMC Psychol. 2024 May 3;12:247. doi: 10.1186/s40359-024-01747-0 (PMC11069255; doi:10.1186/s40359-024-01747-0)
Supplement: Supplementary file 1 — Supplementary Material 1. [file 40359_2024_1747_MOESM1_ESM.docx]

**Online Supplements for**

**Predicting Trajectories of Vocational Indecision from Motivational Profiles in Early Adolescence**

**Authors’ note:**

These online technical appendices are to be posted on the journal website and hot-linked to the manuscript. If the journal does not offer this possibility, these materials can alternatively be posted on one of our websites (we will adjust the in-text reference upon acceptance). We developed these materials to provide additional technical information and to keep the main manuscript from becoming needlessly long.

**Sections**

1. Additional Information for Motivational Profiles in Adulthood
2. Measurement Model and Longitudinal Invariance of Vocational Indecision
3. Covariates Analyses
4. Predictive Similarity and Distribution from Profiles in Indecision Trajectories

# **Section S1. Additional Information for Motivational Profiles in Adulthood**

Some studies focusing on motivation toward sport or work in adult samples and including the amotivation, some of these secondary school and college profiles were obtained^1,2,3,4^. Precisely, the Highly Amotivated profile (very high amotivation and low levels of other types of motivation), the Amotivated profile (high to moderate amotivation, moderate external regulation, and low on other types of motivation), the Controlled-Amotivated profile (high amotivation, moderately high controlled motivations, and low to moderate autonomous motivations), the Low profile (low levels of all motivations), the Moderately or Mixed profile (high to moderate autonomous and controlled motivations and amotivation), the Autonomous-Controlled profile (moderate amotivation and high levels of both autonomous and controlled motivations), the Autonomous-Introjected (moderate autonomous motivations and introjected regulation, low external regulation and very low amotivation^3^ and the Autonomous profile (low amotivation and controlled motivations, and high autonomous motivations). The negative implications of the Highly Amotivated profile seems weaker than those of the Controlled-Amotivated profile^3^.

**Table S1**

*Motivational Profiles in Different Educational Contexts Obtained in Previous Studies*

| Context | Pres.Amo | School  Level and *N* | Analysis | Profile Labels | Characteristics |
| --- | --- | --- | --- | --- | --- |
| Learning^5^ Motivation | No | Secondary  N=178 | LPA | Poor quality | Higher EXT, lower IDEN and INTRIN |
|  |  |  |  | Moderately positive | Highly moderate AUTO and moderate EXT |
|  |  |  |  | Moderately negative | Moderate AUTO and highly moderate EXT |
|  |  |  |  | Good quality | Moderate EXT, highly moderate INTRO, and high AUTO |
| Academic^6^ Motivation | Yes | College  N=510 | Cluster | Additive | High INTRIN, IDEN, INTRO and EXT, low AMO |
|  |  |  |  | SD | Highly moderate INTRIN and IDEN, moderate INTRO and EXT, low AMO |
|  |  |  |  | Moderate | Low AMO and moderate for the others |
|  |  |  |  | Low | Low for all types |
|  |  |  |  | Non-SD | Moderate AUTO and INTRO, high EXT and moderate AMO |
| ^a^Academic^7^ Motivation | Yes | Secondary  N_1_= 210  N_2_= 215 | Cluster | SD | High AUTO, moderate INTRO, low EXT and AMO |
|  |  |  |  | Moderated | Moderate level of all types |
|  |  |  |  | Non-SD | Low AUTO and INTRO, high EXT and AMO |
| Physical^8^ Activity  Motivation | Yes | Secondary  N=181 | LPA | Non-SD | Moderate AUTO, and high CONTR and AMO |
|  |  |  |  | Moderate SD | High AUTO, moderate CONTR, and low AMO |
|  |  |  |  | Moderate | Moderate AUTO, CONTR and AMO |
|  |  |  |  | High SD | High AUTO, low CONTR and AMO |
| Academic^9^ Motivation | No | Secondary  N=388 | Cluster | High quantity | Moderate INTRIN and EXTRIN |
|  |  |  |  | Good Quality | High INTRIN and low EXTRIN |
|  |  |  |  | Poor Quality | Low INTRIN and moderate EXTRIN |
|  |  |  |  | Low quantity | Low INTRIN and EXTRIN |
| Learning ^10^  Motivation | Yes | Secondary  N=767 | Cluster | Low SD-high CONTR | Low AUTO, high CONTR and AMO |
|  |  |  |  | SD-Low CONTR | High AUTO, low CONTR and AMO |
|  |  |  |  | Low SD-Low CONTR | Low AUTO, CONTR and AMO |
|  |  |  |  | High SD-High CONTR | High AUTO and CONTR, and low AMO |
| Academic Motivation^11^ | Yes | Secondary  N= 738 | Cluster | High Quantity | High AMO, moderate AUTO and CONTR |
|  |  |  |  | High Quality | Low AMO, highly moderate AUTO and CONTR |
|  |  |  |  | Low Quantity | Low levels of all |
|  |  |  |  | Low Quality | Moderate AMO, very low AUTO and CONTR |
| Physical^12^ Activity Motivation | Yes | Secondary  N=413 | Cluster | SD | High INTRIN, moderate IDEN, INTRO, EXT and low AMO |
|  |  |  |  | Low SD - Non-SD | Low INTRIN, IDEN, INTRO, EXT and AMO |
|  |  |  |  | Non-SD | Moderate INTRIN and IDEN, low INTRO and EXT |
| Academic Motivation^13^ | No | College  N=396 | Cluster | SD | High INTRIN and IDEN, low INTRO and EXT |
|  |  |  |  | Externally regulated | High INTRIN and IDEN, moderate INTRO and EXT |
|  |  |  |  | Non-SD | Moderate INTRIN and IDEN, low INTRO and EXT |
| ^a^Academic^14^Motivation | Yes | Secondary  N_1_ = 4498  N_2_ = 942 | LPA | Controlled | High to moderate AMO, moderate EXT and IDEN, low INTRIN and INTRO |
|  |  |  |  | Moderate A-C | Moderate AMO, CONTR and AUTO |
|  |  |  |  | High A-C | High to moderate CONTR and AUTO, low AMO |
| Responsible drinking  Motivation^15^ | Yes | College  N=507 | LPA | High Quality | High AUTO and INTRO, low EXT and AMO |
|  |  |  |  | High Quantity | High AUTO, INTRO and AMO, moderate EXT, |
|  |  |  |  | Low Quantity | Low for all |
| Academic Motivation^16^ | No | Secondary  N=386 | Cluster | Average | Weakly moderate AUTO and INTRO, highly moderate EXT |
|  |  |  |  | Motivated | High AUTO and INTRO, moderate EXT |
|  |  |  |  | SD | High AUTO, moderate INTRO, and low EXT |
|  |  |  |  | Low motivation | Low of all types |
| Academic Motivation^17^ | No | College  N=876 | Cluster | Good quality | High AUTO and low CONTR |
|  |  |  |  | High quantity | High AUTO and CONTR |
|  |  |  |  | Poor quality | High CONTR and AUTO |
|  |  |  |  | Low quantity | Low all motivations |
| Learning  Motivation^18^ | No | Secondary  N=342 | LPA | Good Quality | High AUTO, moderate INTRO, and low EXT |
|  |  |  |  | Moderately positive | Moderate AUTO and highly moderate EXT |
|  |  |  |  | Moderately negative | Moderate EXT and highly moderate INTRO and high AUTO |
|  |  |  |  | Poor Quality | Moderate EXT and lower IDEN and INTRIN |
| Academic Motivation^19^ | No | College  N=1066 | Cluster | High quantity | High for all |
|  |  |  |  | Good quality | High INTRIN, moderate INTRO and EXT |
|  |  |  |  | Poor quality | Moderate INTRIN and INTRO and high EXT |
|  |  |  |  | Low quantity with poor quality | Low INTRIN and INTRO and high EXT |

*Note*. ^a^Two different studies. LPA = latent profile analyses. Pres. Amo = inclusion of the amotivation. Types of motivations: AUTO = autonomous; INTRIN= intrinsic; IDEN = identified regulation; CONTR = controlled; INTRO = introjected regulation; EXT= external regulation. AMO= amotivation. SD = self-determined. A-C= autonomous and controlled motivations

# **Section S2. Measurement Model and Longitudinal Invariance of Vocational Indecision**

The CDP measurement model of vocational indecision contained six first-order latent factors representing dimensions of vocational indecision (*Decidedness, Comfort, Self-Clarity, Knowledge about Occupations and Training, Decisiveness, and Career Choice Importance*). In this study, the *Comfort* subscale has been removed because its two items presented very weak factor loadings, in addition to having contrasting valence (negative and positive). As one of its two items loaded very well with the Decidedness subscale, we allowed it to load on this dimension: so only five of the six subscales were used. All first-order latent factors loaded on a second-order latent factor representing vocational indecision^20^. So, because vocational indecision is a multidimensional construct, solutions from confirmatory factorial analysis (CFA) and exploratory structural modeling (ESEM)^21^ were compared. Precisely, we tested and compared four models: two CFA (second-order CFA model [M_1_] and bifactor CFA model [M_2_]) and two ESEM (second order ESEM-within-CFA model [M_3_] and bifactor ESEM-within-CFA model [M_4_]). The ESEM-within-CFA (EWC)^22^ is a hierarchical ESEM in which the CFA framework of an ESEM model is re-expressed by using the loadings generated from the initial ESEM model and where the first-order latent factors have all items loading on all factors (i.e., cross-loadings permitted)^21^. The bifactor models (M_2_ and M_4_) included a general vocational indecision factor on which all items loaded and five specific factors, each corresponding to the five dimensions.

The results showed that even if the ESEM bifactor model (BI-ESEM) was the best-fitting model, its solution included negative residual variances for two items. The same observation was made for the CFA second-order model, even after setting these variances at 0 (i.e., considering that all variance for these items contributed to the general indecision factor). Finally, we chose the EWC model, which presented good fit indices, satisfying factor loadings, and no improper estimates. These results are reported in Table S2. After selecting the measurement model, we tested four longitudinal measurement invariance models, defined below^20^ (see Table S3): (1) configural invariance (i.e., invariance in number of factors and their correspondence with respective items remains stable across waves), (2) metric invariance (i.e., stability of factors loadings and latent factors), scalar invariance (i.e., stability of intercepts of latent factors and the means of observed variables),and residual invariance (most constrained model; i.e., stability of residual variances and covariances for items, or whether they measure the same factors with the same degrees of precision over time).

**Table S1**

*Fit Indices for Factorial Analyses Models of Vocational Indecision (N=384)*

|  | TLI | CFI | RMSEA | [90% CI] | SRMR |
| --- | --- | --- | --- | --- | --- |
| Time 1 |  |  |  |  |  |
| M_1_ | .90 | .88 | .08 | [.07,.09] | .09 |
| M_2_ | .99 | .98 | .03 | [.02,.04] | .03 |
| M_3_ | .97 | .99 | .05 | [.03,.04] | .03 |
| M_4_ | 1.00 | 1.00 | .00 | [.00,.01] | .01 |
| Time 2 |  |  |  |  |  |
| M_1_ | .91 | .88 | .09 | [.07,.09] | .10 |
| M_2_ | .97 | .95 | .05 | [.03,.04] | .97 |
| M_3_ | .98 | 1.00 | .03 | [.02,.04] | .02 |
| M_4_ | .99 | .98 | .04 | [.02,.04] | .01 |
| Time 3 |  |  |  |  |  |
| M_1_ | .91 | .89 | .09 | [.08,.09] | .08 |
| M_2_ | .97 | .94 | .06 | [.05,.07] | .03 |
| M_3_ | .98 | 1.00 | .03 | [.03,.04] | .02 |
| M_4_ | .99 | .96 | .05 | [.03,.06] | .01 |
| Time 4 |  |  |  |  |  |
| M_1_ | .91 | .89 | .10 | [.09,.10] | .08 |
| M_2_ | .96 | .93 | .07 | [.06,.08] | .05 |
| M_3_ | .98 | .98 | .07 | [.06,.08] | .02 |
| M_4_ | 1.00 | 1.00 | .00 | [.00,.01] | .01 |

*Note.* M_1-4_= Models 1 to 4

**Tableau S2**

*Model Fit Indices for Models Evaluating Measurement Longitudinal Invariance of Vocational Indecision (N=384)*

|  | χ^2^ | df | TLI | CFI | RMSEA | SRMR | ∆χ^2^_SB_ |  | ∆df | $\Delta TLI$ | $\Delta CFI$ | $\Delta RMSEA$ | $\Delta SRMR$ |
| --- | --- | --- | --- | --- | --- | --- | --- | --- | --- | --- | --- | --- | --- |
| Configural (M_5_) | 2116.10 | 1406^*^ | .94 | .94 | .02 | .06 | n/a |  |  |  |  |  |  |
| Metric (M_6_) | 2123.95 | 1421^*^ | .94 | .94 | .03 | .06 | M_6_-M_5_ | 12.69 | 15 | .00 | .00 | .01 | .01 |
| Scalar (M_7_) | 2595.10 | 1466^*^ | .90 | .90 | .04 | .08 | M_7_- M_6_ | 492.00 | 45^*^ | .04 | .04 | .01 | .01 |
| Residuals (M_8_) | 2920.25 | 1629^*^ | .89 | .89 | .04 | .08 | M_8_- M_7_ | 144.86 | 43^*^ | .01 | .01 | .00 | .00 |

*Note.* ^*^ *p* < .05. n/a = not applicable; Df = degree of freedom; χ^2^_SB_ = Satorra-Bentler Scaled Chi-Squared Test.

# **Section 3. Covariates Analyzes**

A covariate is a variable that potentially has a predictive effect on a dependent variable so that its inclusion makes it possible to optimize the generalization of the effects of other explanatory variables involved in the predictive model of this dependent variable^20^. The addition of a covariate in an LPA or GGMA can depend on the case, modify the number of latent trajectory classes or profiles. Specifically, the addition of time-varying covariate as predictor could modify the number of latent groups (i.e., trajectories or profiles) obtained from the unconditional model (i.e., the model without any predictor). However, whether stable or not, a covariate could affect the group membership; thus, it is recommended not to include covariate as a predictor in an LPA or GGMA model, but rather as an auxiliary variable^23,24^.

**Auxiliary Approach**

A variable could be a *predictor* if it influences the group membership, *outcome* if it is influenced by the latent group, and *correlate* if there is not any influence link between both^24^. Among the auxiliary analyses, the BCH^25^ and the R3-STEP methods^26^ have been recommended rather than post-hoc analyses. The BCH method conducts a weighted multiple-group ANOVA on differences in means for continuous outcomes, where the weights are a function of the classification probabilities^25^. Here, participants' class probabilities (obtained from unconditional LPA or GGMA solutions) are used to specify their probability of membership in each latent group, so that the group classification includes individual rather than average uncertainty^25^. This method is recommended when testing variable as an outcome of profile or class membership and its main limitation is its underperformance when the entropy is low.

As its name indicates, the R3-STEP method consists of three steps^26^: after the optimal unconditional LPA or GGMA model was estimated and the modal profile membership (i.e., a nominal variable representing a class) saved from it (step 1), a new latent group (i.e., a class or profile) solution is estimated from this modal profile membership (step 2). The classification in the new profiles or latent classes considers the uncertainty of the classification of the initial unconditional model and its based-probability classification. These probabilities are used to specify the probability of membership into each latent profile or class so that the classification includes individual rather than average uncertainty^26^. Finally, step 3 consists of using this nominal variable-based solution in subsequent analyses. This method is recommended when the auxiliary variable is tested as a predictor of the profile or class but does not always completely prevent shifts in the definition of the profiles or classes^26^. The R3-STEP uses logistic regression to calculate odd ratios, allowing to check if auxiliary variables reduce or improve the risk of belonging to a class.

Here, we used the BCH method to compare the estimated motivational profiles and vocational indecision trajectory classes, according to student gender and SAI. The R3-STEP was also used, where the aim was to determine if gender or SAI increased or decreased the probability of belonging to one group more than another, and consequently, whether it needed to be included as predictors of latent groups in the prediction model.

**Results of Auxiliary Analyses**

***Motivational Profiles*.** Adding gender and the SAI to the optimal LPA as outcomes (i.e., using the BCH method), the group proportions slightly changed as follows: Highly Amotivated (5%), Controlled-Amotivated (11%), Mixed (61%), And Autonomous-Introjected (23%). We also found that the Mixed and Autonomous-Introjected profiles included more girls than the two others, and that the SAI was lowest in the Autonomous-Introjected profile and highest Highly Amotivated and Controlled-Amotivated profiles (see Table S4). Also, their inclusion as predictors (i.e., using the R3-STEP method) showed that both affected student profile membership: being a girl decreased the probability of belonging to the Highly Amotivated or Controlled-Amotivated profiles, compared to the Autonomous-Introjected or Mixed profiles. Also, the SAI decreased the probability of being in the Mixed or Autonomous-Introjected profiles, compared to the Controlled-Amotivated profiles (see Table S6).

***Vocational Indecision Trajectories Classes*.** Adding gender and SAI as an outcome in the 4-groups optimal GGMA-LV, we found that the Developmental trajectory included more girls and the lowest family adversity (see Table S5). In addition, being a girl decreased the probability of following the Low and Stable trajectory compared to the Developmental trajectories, and the SAI increased the probability of following Chronic Intermittent, Moderate and Stable, and Low and Stable trajectories, compared to the Developmental trajectory (see Table S6). When both were added as control variables, the group proportions slightly changed as follows: Moderate and Stable (23%), Low and Stable (53%), Developmental (8%), and Chronic Intermittent (15%).

In the unconditional GGMA-LV, the slopes were negative for the Chronic Intermittent trajectory and positive for the Developmental trajectory (see Table S6), suggesting respectively that the decreasing and increasing in the expression of vocational indecision through the secondary school. These slopes and intercepts were weakly and negatively correlated in both Chronic Intermittent and Developmental trajectories (see Table S6). This result suggests that the developmental trend of vocational indecision in secondary school is inversely associated with the initial level of indecision at the beginning of the secondary school (i.e., the lower indecision was in Secondary 2, the more it will increase during the three subsequent school years; and the higher students’ indecision was at T1, the more it decreased in subsequent years).

However, the control GGMA-LV presented some minor differences: the Chronic Intermittent trajectory exhibited a higher intercept, a slightly steeper downward slope, but its quadratic effect appeared unchanged (see Table S7). This suggested that when gender and family adversity were considered, students with a Chronic Intermittent trajectory were even more undecided in Secondary 2. Also, the Developmental trajectory showed smaller intercept, slope, and quadratic effect (see Table S7), suggesting that when controlling for gender and familial adversity, students in a Developmental trajectory were even less undecided in Secondary 2.

**Table S3**

*Equality Tests of Means for Control Variables Across Motivational Profiles at Secondary 2*

|  | Motivational profiles | | | |  |
| --- | --- | --- | --- | --- | --- |
|  | P1 | P2 | P3 | P4 | Groups comparisons |
| Gender^a^ | 1.60 (.04) | 1.33 (.11) | 1.37 (.08) | 1.60 (.06) | P2 = P3 < P1 = P4 |
| SAI | .24 (.02) | .28 (.07) | .36 (.06) | .21 (.03) | P4 < P1< P2 = P3 |

*Note*. P1 = Mixed. P2 = Highly Amotivated. P3 = Controlled-Amotivated. P4 = Autonmous-Introjected. ^a^ 1= boy, 2= girl.

**Table S4**

*Equality Tests of Means for Control Variables Across Classes of Vocational Indecision Trajectories*

|  | Trajectories of vocational indecision | | | |  |
| --- | --- | --- | --- | --- | --- |
|  | C1 | C2 | C3 | C4 | Groups comparisons |
| Gender^a^ | 1.57 (.06) | 1.73 (.09) | 1.51 (.04) | 1.59 (.08) | C3 < C4 = C1 = C2 |
| SAI | .30 (.04) | .12 (.04) | .25 (.02) | .26 (.05) | C4 < C3 = C2< C1 |

*Note*. C1 = Moderate and stable. C2 = Developmental. C3 = Low and Stable. C4 = Chronic Intermittent. ^a^ 1= boy, 2= girl.

**Table S5**

*Odds Ratios for Tests of Gender and SAI Multinomial Logistic Regressions among Vocational Indecision Trajectories and Motivational Profiles (N=384)*

|  | Gender | | |  | SAI | | |
| --- | --- | --- | --- | --- | --- | --- | --- |
| Groups Comparisons | OR | (SE) | *p* |  | OR | (SE) | *p* |
| Vocational Indecision Trajectories |  | | |  |  | | |
| Moderate and Stable (*vs* Developmental) | .52 | (.26) | .06 |  | 9.41 | (7.68) | .27 |
| Chronic Intermittent (*vs* Developmental) | .57 | (.31) | .16 |  | 6.23 | (5.52) | .34 |
| Low and Stable (*vs* Developmental) | **.40** | **(.19)** | **.00** |  | 5.88 | (4.66) | .30 |
| Chronic Intermittent (*vs* Moderate and Stable) | 1.09 | (.44) | .83 |  | .66 | (.41) | .41 |
| Low and Stable (*vs* Moderate and Stable) | .78 | (.25) | .37 |  | .63 | (.31) | .22 |
| Developmental (*vs* Moderate and Stable) | 1.93 | (.97) | .34 |  | **.11** | **(.09)** | **.00** |
| Low and Stable (*vs* Chronic Intermittent) | .71 | (.27) | .28 |  | .94 | (.55) | .92 |
| Moderate and Stable (*vs* Chronic Intermittent) | .92 | (.37) | .82 |  | 1.51 | (.93) | .58 |
| Developmental (*vs* Chronic Intermittent) | 1.77 | (.97) | .42 |  | **.16** | **(.14)** | **.00** |
| Chronic Intermittent (*vs* Low and Stable) | 1.40 | (.52) | .44 |  | 1.06 | (.62) | .92 |
| Moderate and Stable (*vs* Low and Stable) | 1.28 | (.40) | .49 |  | 1.60 | (.78) | .44 |
| Developmental (*vs* Low and Stable) | 2.48 | (1.19) | .21 |  | **.17** | **(.14)** | **.00** |
| Motivational Profiles |  | | |  |  | | |
| Mixed (*vs* Autonomous-Introjected) | 1.01 | (.33) | .97 |  | 1.47 | (.78) | .55 |
| Highly Amotivated (*vs* Autonomous-Introjected) | **.34** | **(.19)** | **.00** |  | 2.10 | (1.74) | .53 |
| Controlled-Amotivated (*vs*autonomous-Introjected) | **.39** | **(.16)** | **.00** |  | 4.56 | (2.82) | .21 |
| Mixed (*vs* Controlled-Amotivated) | 2.60 | (.98) | .10 |  | **.32** | **(.18)** | **.00** |
| Highly Amotivated (*vs* Controlled-Amotivated) | .87 | (.54) | .81 |  | .46 | (.39) | .16 |
| Autonomous-Introjected (*vs* Controlled-Amotivated) | 2.57 | (1.08) | .14 |  | **.22** | **(.14)** | **.00** |
| Mixed (*vs* Highly Amotivated) | 3.00 | (1.59) | .21 |  | .70 | (.53) | .57 |
| Controlled-Amotivated (*vs* Highly Amotivated) | 1.15 | (.71) | .83 |  | 2.17 | (1.81) | .52 |
| Autonomous-Introjected (*vs* Highly Amotivated) | 2.96 | (1.68) | .24 |  | .48 | (.39) | .18 |
| Highly Amotivated (*vs* Mixed) | **.33** | **(.18)** | **.00** |  | 1.43 | (1.09) | .69 |
| Controlled-Amotivated (*vs* Mixed) | **.39** | **(.15)** | **.00** |  | 3.10 | (1.70) | .22 |
| Autonomous-Introjected (*vs* Mixed) | .99 | (.32) | .97 |  | .68 | (.36) | .37 |

*Note*. OR = odd ratio. SE = standard error. p = *p* value. Bolded coefficients represent statistically significant estimates.

**Table S6**

*Functional Shapes for the Unconditional GGMA-LV and Control GGMA-LV Trajectories (N=384)*

| Functional Shapes | I | | | S | | | Q | | | I with S | | |
| --- | --- | --- | --- | --- | --- | --- | --- | --- | --- | --- | --- | --- |
|  | M | SE | *p* | M | SE | *p* | M | SE | *p* | *r* | SE | *p* |
| Unconditional GGMA-LV |  |  |  |  |  |  |  |  |  |  |  |  |
| Moderate and Stable | .00 | .01 | .90 | -.01 | .02 | .51 | .01 | .01 | .32 | .00 | .00 | ‒ |
| Chronic intermittent | .44 | .14 | .00 | -.70 | .16 | .00 | .24 | .05 | .00 | -.18 | .04 | .00 |
| Developmental | -.38 | .18 | .03 | 1.23 | .19 | .00 | -.40 | .05 | .00 | -.13 | .02 | .00 |
| Low and Stable | -.17 | .05 | .00 | .06 | .04 | .13 | -.02 | .01 | .14 | -.02 | .01 | .00 |
| Control GGMA-LV | | | | | | | | | | | | |
| Moderate and Stable | .00 | .01 | .85 | -.01 | .01 | .54 | .01 | .01 | .32 | .00 | .00 | ‒ |
| Chronic intermittent | .53 | .24 | .03 | -.79 | .32 | .01 | .26 | .09 | .00 | -.16 | .05 | .00 |
| Developmental | -.42 | .17 | .01 | 1.10 | .34 | .00 | -.35 | .11 | .00 | -.17 | .04 | .00 |
| Low and Stable | -.15 | .06 | .01 | .04 | .05 | .48 | -.01 | .02 | .53 | -.02 | .01 | .01 |

*Note*. I = intercept. S = slope. Q = quadratic. I with S = correlation between intercept and slope.

# **Section S4. Predictive LTA**

**Model Estimation**

The LTA presented two components: the first was the growth mixture part, corresponding to the optimal 4-groups GGMA-LV solution, and the second was the LPA part, corresponding to the optimal solution for the 4-groups motivational profiles at T1^24^. So, the prediction of vocational indecision trajectory membership (growth mixture part) by motivational profiles (LPA part) was done by regressing trajectory membership of *N-1* classes (i.e., 3 classes) of vocational indecision in reference to the *N^th^* class (i.e., the fourth or reference), on *Z-1* motivational profiles (i.e., 3 profiles) membership in reference to the *Z^th^* profile (i.e., the fourth or reference)^24^. In this way, several LTA models were tested, depending on the reference trajectory class or profile, and using the starting values from the LTA model without any predictor.

Results of students’ distribution into the four profiles corroborate that of multinomial analyses. Precisely, students were unequally distributed in the different trajectories (see Figure S1). The Low and Stable trajectory was followed by the larger proportion of each profile: nearly 60% for the Controlled-Amotivated and Mixed profiles, 50% for the Highly Amotivated profile, and 42% for the Autonomous-Introjected profile. The Moderate and Stable trajectory was followed mainly by individuals with an Autonomous-Introjected profile (33%), followed by Controlled-Amotivated (27%), Highly Amotivated (28%), and the Mixed profile (18%). None of the students from the Highly Amotivated profile followed the Developmental trajectory, which mostly included students with an Autonomous-Introjected profile (17%) and much fewer from Controlled-Amotivated and Mixed profiles (9% and 6%, respectively). Finally, the Chronic Intermittent trajectory was predominantly followed by students with a Highly Amotivated profile (22%), seconded by those with the Mixed profile (18%). Students belonging to the Autonomous-Introjected or Controlled-Amotivated profiles followed this trajectory less, with respectively 8% and 6% of their students.

**Figure S1**

*Distribution of Students from the Four Motivational Profiles into Vocational Indecision Trajectories (N=384)*

*
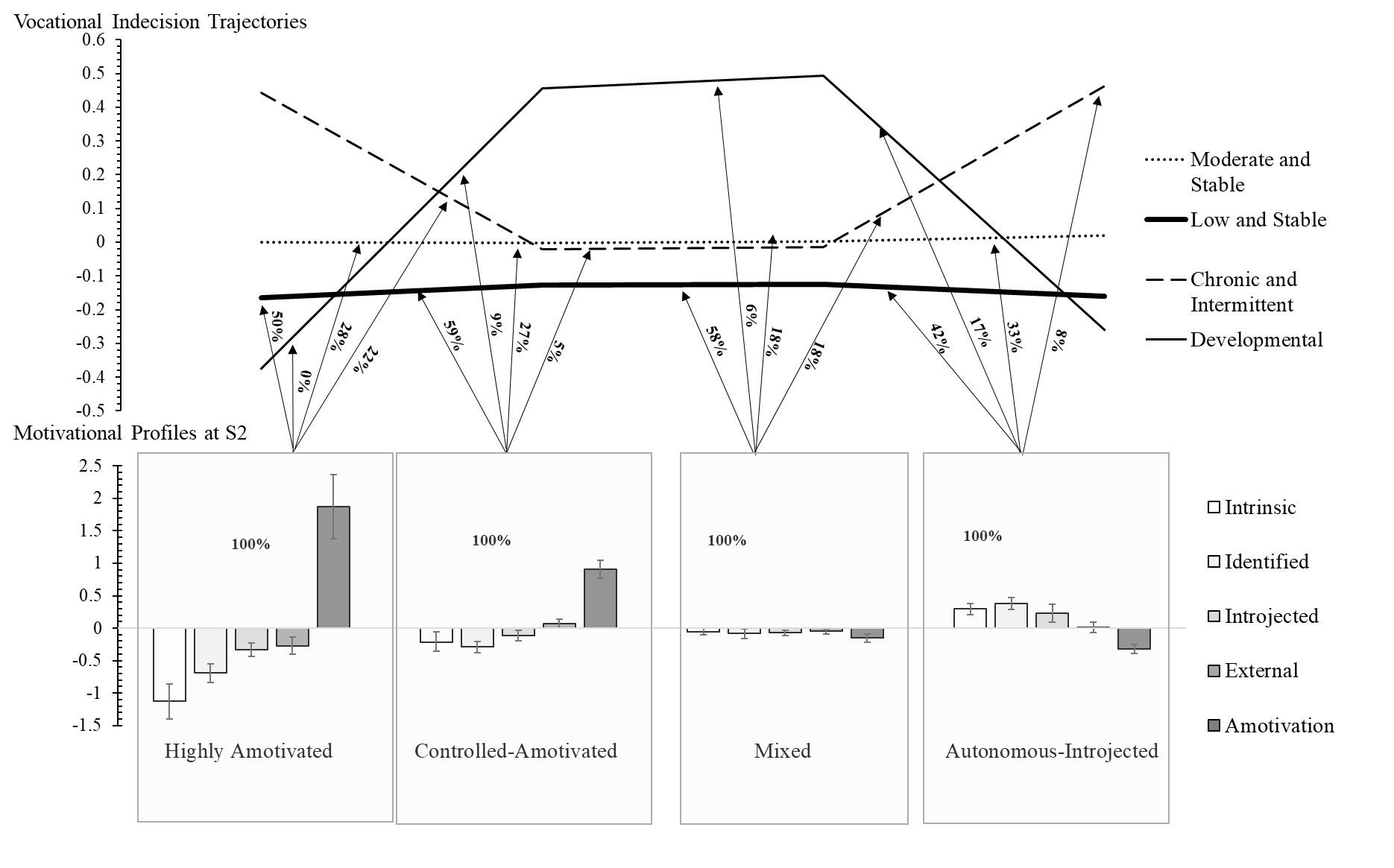
*

**References**

1. Emm-Collison, L. G., Sebire, S. J., Salway, R., Thompson, J. L., & Jago, R. (2020). Multidimensional motivation for exercise: A latent profile and transition analysis. *Psychology of Sport and Exercise*, 47, 101619.
2. Fernet, C., Litalien, D., Morin, A. J. S., Austin, S., Gagné, M., Lavoie-Tremblay, M., & Forest, J. (2020). On the temporal stability of self-determined work motivation profiles : A latent transition analysis. *European Journal of Work and Organizational Psychology*, *29*(1), 49‑63. <https://doi.org/10.1080/1359432X.2019.168830>
3. Gustafsson, H., Carlin, M., Podlog, L., Stenling, A., & Lindwall, M. (2018). Motivational profiles and burnout in elite athletes: A person-centered approach. *Psychology of Sport and Exercise*, 35, 118‑125.
4. Morbée, S., Haerens, L., Waterschoot, J., & Vansteenkiste, M. (2021). Which cyclists manage to cope with the corona crisis in a resilient way? The role of motivational profiles. *International Journal of Sport and Exercise Psychology*, 1‑19. <https://doi.org/10.1080/1612197X.2021.1940241>
5. Baars, M., & Wijnia, L. (2018). The relation between task-specific motivational profiles and
6. training of self-regulated learning skills. *Learning and Individual Differences*, 64
7. Boiché, J., & Stephan, Y. (2014). Motivational profiles and achievement: A prospective study testing potential mediators. *Motivation and Emotion*, *38*(1), 79‑92. <https://doi.org/10.1007/s11031-013-9361-6>
8. Boiché, J. C. S., Sarrazin, P. G., Grouzet, F. M. E., Pelletier, L. G., & Chanal, J. P. (2008). Students’ motivational profiles and achievement outcomes in physical education : A self-determination perspective. *Journal of Educational Psychology*, 100(3), 688‑701. <https://doi.org/10.1037/0022-0663.100.3.688>
9. Guijarro-Romero, S., Mayorga-Vega, D., Casado-Robles, C., & Viciana, J. (2020). Does students’ self-determined motivation toward Physical Education influence the effectiveness of a fitness teaching unit? A cluster-randomized controlled trial and cluster analysis. *Psychology of Sport and Exercise*, *51*, 101768. <https://doi.org/10.1016/j.psychsport.2020.101768>
10. Hayenga, A. O., & Corpus, J. H. (2010). *Proﬁles of intrinsic and extrinsic motivations: A person-centered approach to motivation and achievement in middle school*. 13.
11. Liu, W. C., Wang, C. K. J., Tan, O. S., Koh, C., & Ee, J. (2009). A self-determination approach to understanding students’ motivation in project work. *Learning and Individual Differences*, *19*(1), 139‑145.
12. Manzano-Sánchez, D., Gómez-Marmol, A., Jiménez-Parra, J. F., Bohórquez, I. G., & Valero-Valenzuela, A. (2021). Motivational profiles and their relationship with responsibility, school social climate and resilience in high school students. *PLOS ONE*, *16*(8), e0256293. https://doi.org/10.1371/journal.pone.0256293
13. Moreno-Murcia, J. A., & Corbí, M. (2021). Social support by teacher and motivational profile of Higher Education students. Psychology, Society & Education, 13(1), 9‑25. <https://doi.org/10.25115/psye.v1i1.2658>
14. Paixão, O., & Gamboa, V. (2017). Motivational Profiles and Career Decision Making of High School Students. *Career Development Quarterly*, *65*(3), 207‑221. <https://doi.org/10.1002/cdq.12093>
15. Ratelle, C. F., Guay, F., Vallerand, R. J., Larose, S., & Senécal, C. (2007). Autonomous, controlled, and amotivated types of academic motivation : A person-oriented analysis. Journal of Educational Psychology, 99(4), 388‑400. <https://doi.org/10.1016/j.cedpsych.2014.09.003>
16. Richards, D. K., Pearson, M. R., & Field, C. A. (2020). Profiles of motivations for responsible drinking among college students: A self-determination theory perspective. *Addictive Behaviors*, *111*, 106550. <https://doi.org/10.1016/j.addbeh.2020.106550>
17. Ullrich-French, S., & Cox, A. (2009). Using Cluster Analysis to Examine the Combinations of Motivation Regulations of Physical Education Students. *Journal of Sport and Exercise Psychology*, *31*(3), 358‑379. <https://doi.org/10.1123/jsep.31.3.358>
18. Vansteenkiste, M., Smeets, S., Soenens, B., Lens, W., Matos, L., & Deci, E. L. (2010). Autonomous and controlled regulation of performance-approach goals: Their relations to perfectionism and educational outcomes. Motivation and Emotion, 34(4), 333‑353. <https://doi.org/10.1007/s11031-010-9188-3>
19. Wijnia, L., & Baars, M. (2021). The role of motivational profiles in learning problem-solving and self-assessment skills with video modeling examples. *Instructional Science*, *49*(1), 67‑107. <https://doi.org/10.1007/s11251-020-09531-4>
20. Wormington, S., Corpus, J., & Anderson, K. (2012). A person-centered investigation of academic motivation and its correlates in high school. *Learning and Individual Differences*, *22*, 429‑438. <https://doi.org/10.1016/j.lindif.2012.03.004>
21. Kline, R. B. (2015). *Principles and Practice of Structural Equation Modeling, Fourth Edition*. Guilford Publications
22. Marsh, H. W., Morin, A. J. S., Parker, P. D., & Kaur, G. (2014). Exploratory Structural Equation Modeling: An Integration of the Best Features of Exploratory and Confirmatory Factor Analysis. *Annual Review of Clinical Psychology*, *10*(1), 85‑110. <https://doi.org/10.1146/annurev-clinpsy-032813-153700>
23. Morin, A. J. S., & Asparouhov, T. (2018). *Estimation of a Hierarchical Exploratory Structural Equation Model (ESEM) Using ESEM-within-CFA*. Substantive Methodological Synergy Research Laboratory; /z-wcorg/.
24. Hong, W., Bernacki, M. L., & Perera, H. N. (2020). A latent profile analysis of undergraduates’ achievement motivations and metacognitive behaviors, and their relations to achievement in science. *Journal of Educational Psychology*, *112*(7), 1409‑1430. <https://doi.org/10.1037/edu0000445>
25. Morin, A. J. S., McLarnon, M. J. W., & Litalien, D. (2020). Mixture modeling for organizational behavior research. Dans Y. Griep & S. Hansen, *Handbook on the Temporal Dynamics of Organizational Behavior* (p. 351‑379). Edward Elgar Publishing. <https://doi.org/10.4337/9781788974387.00031>
26. Bakk, Z., & Vermunt, J. K. (2016). Robustness of Stepwise Latent Class Modeling with Continuous Distal Outcomes. *Structural Equation Modeling: A Multidisciplinary Journal*, *23*(1), 20‑31. <https://doi.org/10.1080/10705511.2014.95510>
27. Asparouhov, T., & Muthén, B. (2014). Auxiliary Variables in Mixture Modeling: Three-Step Approaches Using M *plus*. *Structural Equation Modeling: A Multidisciplinary Journal*, *21*(3), 329‑341. <https://doi.org/10.1080/10705511.2014.9151814>
